# Gene expression profiles between cystic and solid vestibular schwannoma indicate susceptible molecules and pathways in the cystic formation of vestibular schwannoma

**DOI:** 10.1007/s10142-019-00672-5

**Published:** 2019-04-05

**Authors:** Shuang Yan, Quan Wang, Zirong Huo, Tao Yang, Xiaoling Yin, Zhaoyan Wang, Zhihua Zhang, Hao Wu

**Affiliations:** 1 Department of Otorhinolaryngology, Head & Neck Surgery, Shanghai Ninth People’s Hospital, Shanghai Jiaotong University School of Medicine, Shanghai, China; 2 Ear Institute, Shanghai Jiaotong University School of Medicine, Shanghai, China; 3 Shanghai Key Laboratory of Translational Medicine on Ear and Nose Diseases, Shanghai, China; 4 Department of Otolaryngology & Head and Neck Surgery, Ruijin Hospital, Shanghai Jiao Tong University School of Medicine, Shanghai, China

**Keywords:** Vestibular schwannoma, Cystic formation, Bioinformatics analysis, Potential biomarker

## Abstract

Previous research has shown that although NF2 gene mutation is the major cause of vestibular schwannoma (VS), it may not directly participate in cystic VS (CVS). To elucidate the underlying potential genetic mechanisms in the cystic formation of VS, we compared differences in gene expression between solid VS (SVS) and CVS via a bioinformatics analysis. The cDNA microarray method and miRNA sequencing were performed on 29 representative VSs (17 CVSs and 12 SVSs). A differential expression analysis was used to identify differentially expressed mRNAs (DEmRNAs) and miRNAs (DEmiRNAs). Then, miRNA–mRNA regulatory networks were constructed. Gene ontology (GO), a KEGG pathway enrichment analysis, and the protein–protein interaction (PPI) were used to analyze the co-differentially expressed DEmRNAs at the functional level. From the differential expression analyses, 1304 DEmRNAs, 55 DEmiRNAs, and hub genes including PTEN, FOXO1, FOXO3, VEGFA, and SIRT1 were identified. Histological evidence is presented to confirm the makeup of the hubs, which corresponded with the cDNA microarray. Our analysis revealed that the maps of apoptosis, cellular response to hypoxia, and the PI3K-Akt, AMPK, FOXO, and chemokine signaling pathways were significantly enriched. In addition, the TUNEL assay, immunoblotting analysis, and transmission electron microscope revealed increased degenerative changes in CVS. These findings could be the foundation for understanding the potential role of differential genes in the cystic formation of VS and be helpful in exploring the potential biomarkers for the differential diagnosis, prognosis, and development of drug targets for CVS.

**Electronic supplementary material:**

The online version of this article (10.1007/s10142-019-00672-5) contains supplementary material, which is available to authorized users.

## Introduction

Vestibular schwannoma (VS) arises from the vestibular branch of the eighth cranial nerve, accounting for 6–8% of all intracranial tumors(Wandong et al. 2005; Piccirillo et al. 2009). Although histologically benign, VS often leads to tinnitus, asymmetric hearing loss, disequilibrium, facial numbness, and facial weakness. If left untreated, these tumors can present with catastrophic complications, such as hydrocephalus, brain stem compression, herniation, and even death.

VS can be classified into two subtypes depending on neuroradiological appearance: solid VS (SVS) and cystic VS (CVS) (Piccirillo et al. 2009). CVS is estimated to constitute 6.8 to 20.4% of VS(Jones et al. 2007; Sinha and Sharma 2008; Piccirillo et al. 2009; Jian et al. 2011) and is characterized by aggressive clinical features, such as a rapid rate of tumor expansion(Selesnick and Johnson 1998), frequent adherence to the facial nerve(de Ipolyi et al. 2008), and unpredictable biological behavior(Moon et al. 2007; Mehrotra et al. 2008). It often presents a predicament for neurotologists in choosing management options for cystic vestibular schwannomas: observation will delay the optimal treatment time due to sudden or persistent fast growth, radiotherapy may increase the risk of rapid cyst expansion (de Ipolyi et al. 2008), and microsurgical resection could achieve poorer postoperative functional outcomes compared with solid lesions(Sinha and Sharma 2008).

Elucidating the underlying potential genetic mechanisms in the cystic formation of VS may be helpful in the treatment of CVS. Mutations in the NF2 gene are essential for VS pathogenesis (Jacoby et al. 1994; Cayé-Thomasen et al. 2010); however, our previous study concluded that dysregulation in the NF2 gene is not directly involved in the cystic formation of VS (Zhang et al. 2014).

In the present study, we aimed to closely analyze the differences in gene profiles between SVS and CVS to identify differentially expressed mRNAs (DEmRNAs), miRNAs (DEmiRNA), and hub genes. DEmRNAs and DEmiRNAs can be used for screening susceptible pathways in cystic formation, and hub genes are supposed to facilitate clinical work after carefully considering clinical relevance.

## Materials and methods

### Patients and tumor tissue samples

Fresh tumor specimens were collected from patients diagnosed with sporadic VS who underwent microsurgery in a general hospital between June 2008 and June 2016. Tumors could be defined as cystic depending on the following appearances (Fig. S1): the presence of hypointense areas on T1-weighted postgadolinium magnetic resonance imaging and intraoperative identification of cystic elements (Benech et al. 2005).

All patients signed informed consent forms releasing a neoplasm sample for research purposes. This study was approved by the Ethics Committee of the Shanghai Jiaotong University School of Medicine, Ninth People’s Hospital, Shanghai, China.

### cDNA microarray analysis and miRNA sequencing

Tumor tissue samples including 11 CVSs and 6 SVSs from the subcapsular part were immediately immersed in the RNA store reagent (Beyotime, Shanghai, China) after resection. Total RNA was extracted using the TRIzol reagent (Invitrogen, CA) and reverse transcribed into complementary DNA (cDNA) using the Quantscript RT kit (Tiangen Biotech). Hybridization was performed overnight on the Illumina Human-12T v4 Expression BeadChip (Illumina, San Diego, CA), which contains 47,231 probes per array product. The data of the gene expression profiles were analyzed using the Illumina BeadStudio module application (Illumina).

miRNA sequencing was performed in 12 tissue samples (6 CVSs and 6 SVSs). The tumor tissues were dipped in the RNAstore Reagent (Beyotime, Shanghai, China) immediately and delivered to Jing Neng Bio-Technology Corporation (Shanghai, China) with dry ice for high-throughput sequencing. The 12-sample sequencing data were combined to construct a miRNA library of VS tissue. By evaluating the number of transcripts per million (TPM), the sequences in each sample were compared with the miRNA library established during the study.

### Differential expression analysis

To identify differentially expressed mRNAs or miRNAs between CVSs and SVSs, differential expression analyses were completed by the Illumina package R data structures, which could be used by many algorithms in other R packages. The distributions of value data could be viewed graphically or exported as a statistical summary table, which is useful for determining if these data are median-centered across samples and thus suitable for cross-comparison. The extreme outliers in the data set were removed by t-distributed stochastic neighbor embedding (t-SNE) (Fig. S2).

A difference of two or greater in the intensity of gene expression between CVSs and SVSs was sought. Genes or miRNAs that were overexpressed or underexpressed were cataloged. A Student’s *t* test was then used to identify DEmRNAs or significantly differentially expressed miRNAs (DEmiRNA), with a *p* value cutoff of < 0.05 and a fold change cutoff at 2. To review the characteristics of the mRNA and miRNA expression profiles, heat maps were further generated by a hierarchical clustering analysis based on the normalized values of DEmRNAs and DEmiRNAs using the pheatmap package in R.

### Correlation and co-expression analysis

The construction of miRNA–mRNA interaction pairs was a better way to understand the functional forecast of miRNAs. To integrate the miRNA and mRNA expression profiles, we combined DEmiRNAs with experimentally verified interactions of mRNA targets from miRTarBase (http://mirtarbase.mbc.nctu.edu.tw/php/index.php). These interactions were supported by strong experimental evidence (reporter assay or western blot). miRNA–mRNA networks were constructed and visualized by Cytoscape (http://cytoscape.org/).

### Delineation of functional annotation

Considering that miRNAs execute functions by regulating the expression of target genes, the biological function of the co-differentially expressed DEmRNAs needs to be identified. As a canonical method to annotate genes and gene products, the gene ontology (GO) analysis, including biological process (BP), cellular component (CC), and molecular function (MF), helps identify biological traits for high-throughput genome or transcriptome data. KEGG (http://www.genome.jp/) is a common omnibus for systematically interpreting gene functions, facilitating an intensive understanding of genomic information and higher-order functional information (Kanehisa and Goto 2000). Feeding a certain gene list to the DAVID database (https://david.ncifcrf.gov/) is an essential procedure for further functional analysis and relevant biological annotation of high-throughput genome or transcriptome data (Dennis et al. 2003). To annotate DEmRNAs at the functional level, GO enrichment and KEGG pathway analyses were performed with the DAVID online tool. Statistically significant *p* values were set at 0.05 or less.

### Apoptosis evaluation by TUNEL assay

The terminal deoxynucleotidyl transferase–mediated dUTP-nick end labeling (TUNEL) assay was performed to identify apoptosis in VS cells. The tumor tissue sections were deparaffinized in xylene and hydrated in alcohol, and then the sections were stained with the In Situ Cell Death Detection Kit (Roche, Mannheim, Germany). The sections were incubated with the tunnel reaction mixture and kept at 37 °C for 2 h in the dark. Then, 4′, 6-diamidino-2-phenylindole (DAPI, CST, US) DAPI was added to label the nuclei. Finally, they were observed with a Nikon H500S electron microscope. The percentage of TUNEL-positive nuclei was assessed from at least 100 randomly selected cells from each of the three different SVS or CVS samples (five slides of each sample for quantitative analysis).

### Immunoblotting analysis

Immunoblotting analysis was performed with antibodies specific for caspase-3 (1;1000, #9662, Cell Signaling), cleaved caspase-3 (1;1000, #9664, Cell Signaling), caspase-9 (1;1000, ab202068, Abcam), and cleaved caspase-9 (1;1000, #52873, Cell Signaling). The β-actin antibody (1;1000, #AA128, Beyotime) was used to ensure equal loading of total protein.

### Transmission electron microscopy

The VS tissues were cut into samples with a size of 1 mm^3^ and rapidly fixed in 2.5% glutaraldehyde. Tumor samples were washed three times with 0.1 M phosphate buffer and then fixed with 1% osmium tetroxide (Pelco, US) at 4 °C. After dehydration with ethanol, the samples were embedded in Spurr resin (SPI-Chem, US) and taken for polymerization and repair. Finally, the embedded blocks were sliced into ultrathin slices, stained with both uranyl acetate (SPI-Chem, US) and lead citrate (SPI-Chem,US), and observed under a transmission electron microscope (JEM1230, JEOL, Japan). All images of ultrathin slices and electron microscope sections shown are representative of at least three individual tissues in each group.

### Protein–protein interaction and module analysis

The Search Tool for the Retrieval of Interacting Genes (STRING) database is an online tool for evaluating protein–protein interaction (PPI) information. The latest version of STRING (version 10.0) covers 184 million interactions of 9.6 million proteins from 2031 organisms. DEmRNAs were processed in STRING to uncover the most significant interactive relationships, in which a criterion of a combined score > 0.4 was set as the significance level. The information on the PPI networks from STRING was analyzed in Cytoscape software, in which a tool called Molecular Complex Detection (MCODE) directly illustrated the most significant clusters of the PPI network. The false degree cutoff, node score cutoff, haircut, false K core, and max depth from seed were set at 2, 0.2, true, 2, and 100, respectively.

### Immunohistochemical evaluation

The tumor tissue sections were dewaxed and rehydrated. An antigen retrieval procedure was performed following immersion in EDTA buffer (pH9.0) and rinsing with PBS (pH7.4). The endogenous peroxidase was then blocked with 3% hydrogen peroxide solution for 25 min at room temperature in the dark, and nonspecific binding was blocked with 3% bull serum albumin (BSA) for 30 min. Then, the sections were serially incubated with anti-PTEN antibody (1:300, GB11169, Servicebio, Wuhan, China), anti-SIRT1 antibody (1:100, GB1171, Servicebio, Wuhan, China), anti-FOXO1 (1:500, GB11286-1, Servicebio, Wuhan, China), anti-FOXO3 (1:200; NBP2-16521, NOVUS, Colorado, USA), and anti-VEGFA (1:300, GB1034, Servicebio, Wuhan, China) at 4 °C for 24 h and then washed in PBS. Next, the sections were incubated for 50 min in goat anti-rabbit/mouse IgG-HRP polymer and then incubated with DAB Chromogen. The percentage of positively stained cells was assessed from at least 100 randomly selected cells from each of the three different SVS or CVS samples (five slides of each sample for quantitative analysis). Finally, the sections were photographed under a light microscope.

## Results

### Data processing of DEmRNAs and DEmiRNAs

To identify the possible gene expressional change in VS, 29 representative tumors (17 CVSs and 12 SVSs) were subjected to a differentially expressed mRNA and miRNA analysis using the cDNA microarray method and high-throughput sequencing, respectively. The t-distributed stochastic neighbor embedding (t-SNE) of the cDNA microarray and miRNA sequencing data demonstrated the global separation of the CVS and SVS samples (Supplementary Fig. 2A and Fig. 2b).

**Fig. 1:**
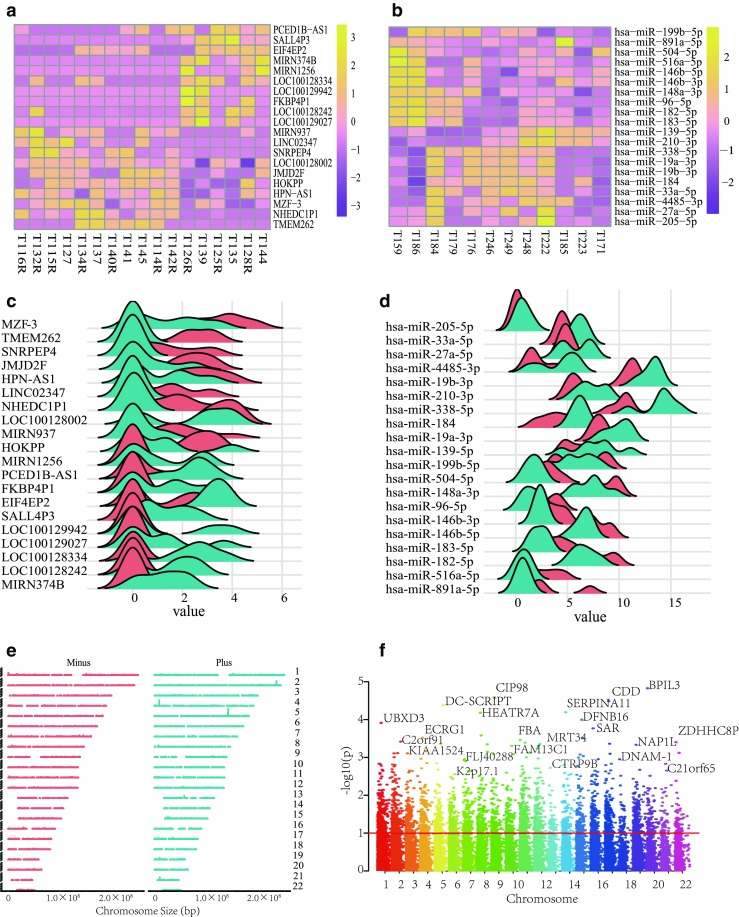
Differentially expressed mRNAs (DEmRNAs), miRNAs (DEmiRNAs), and DEmRNAs’ chromosome locus analysis. **a**, **b** Hierarchical clustering analysis of DEmRNAs and DEmiRNAs for a comparison of CVS and SVS samples. Yellow, higher expression of DEmRNAs or DEmiRNAs; purple, lower expression of DEmRNAs or DEmiRNAs. **c**, **d** The expression of top 10 upregulated and downregulated DEmRNAs and DEmiRNAs. **e**, **f** Peak visualization and Manhattan plots presenting expression levels and *p* values relative to chromosome locus

With the expression level normalized, 1304 mRNAs and 55 miRNAs were found to be differentially expressed between SVSs and CVSs. Among these DEmRNAs, 647 were downregulated and 657 were upregulated. Out of 55 DEmiRNAs, 33 miRNAs were downregulated and 22 miRNAs were upregulated. The top 10 upregulated and downregulated DEmRNAs and DEmiRNAs are shown in Fig. 1a and Fig. 1b. Additionally, Fig. 1c and Fig. 1d present the expression level distributions of these DEmRNAs and DEmiRNAs in CVSs and SVSs.

Plots of the chromosomal locations of the DEmRNAs are shown in Fig. 1e and Fig. 1f. As shown in Fig. 1e, the DEmRNAs were approximately located in the plus strand and minus strand. A total of 34,690 genes were scattered by their *p* values and chromosomes in Fig. 1f. Genetic mapping information from a total of 34,690 genes was scattered by their *p* values and chromosomes. The most significant mRNAs of all chromosomes were associated with the negative log of the corresponding *p* value between 0.01 and 0.001, as highlighted in Fig. 1e.

### Co-expression of miRNAs and mRNAs in CVS

**Fig. 2:**
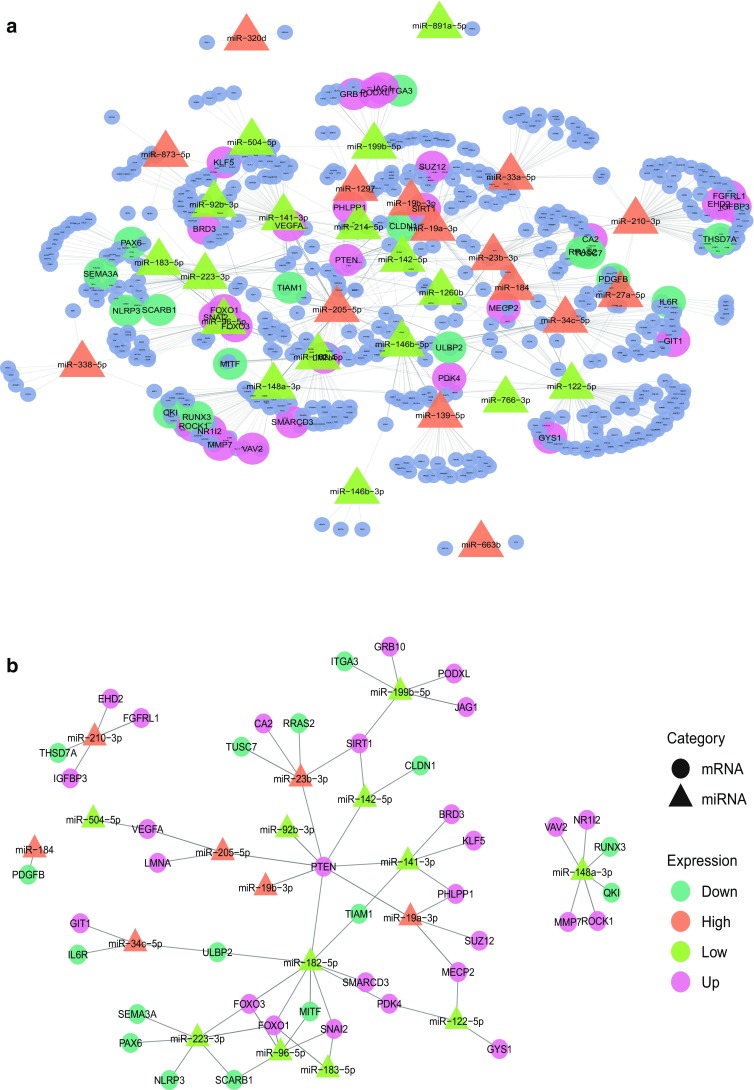
Networks of DEmiRNAs and DEmRNAs. Circle nodes represent mRNAs and triangle nodes represent miRNAs, and their interactions were represented by gray edges

Considering that miRNAs execute functions by regulating the expression of target genes, we combined all 55 DEmiRNAs with experimentally verified interactions of mRNA targets from miRTarBase. Out of these targets, 540 were predicted mRNAs; however, only 43 were on the DEmRNAs list (Fig. 2a). We assumed that these 55 DEmiRNAs and 43 DEmRNAs may contribute to the cystic formation. Figure 2b shows the 67 interaction pairs between these DEmiRNAs and DEmRNAs.

### Biofunction enrichment analysis and verification

**Fig. 3:**
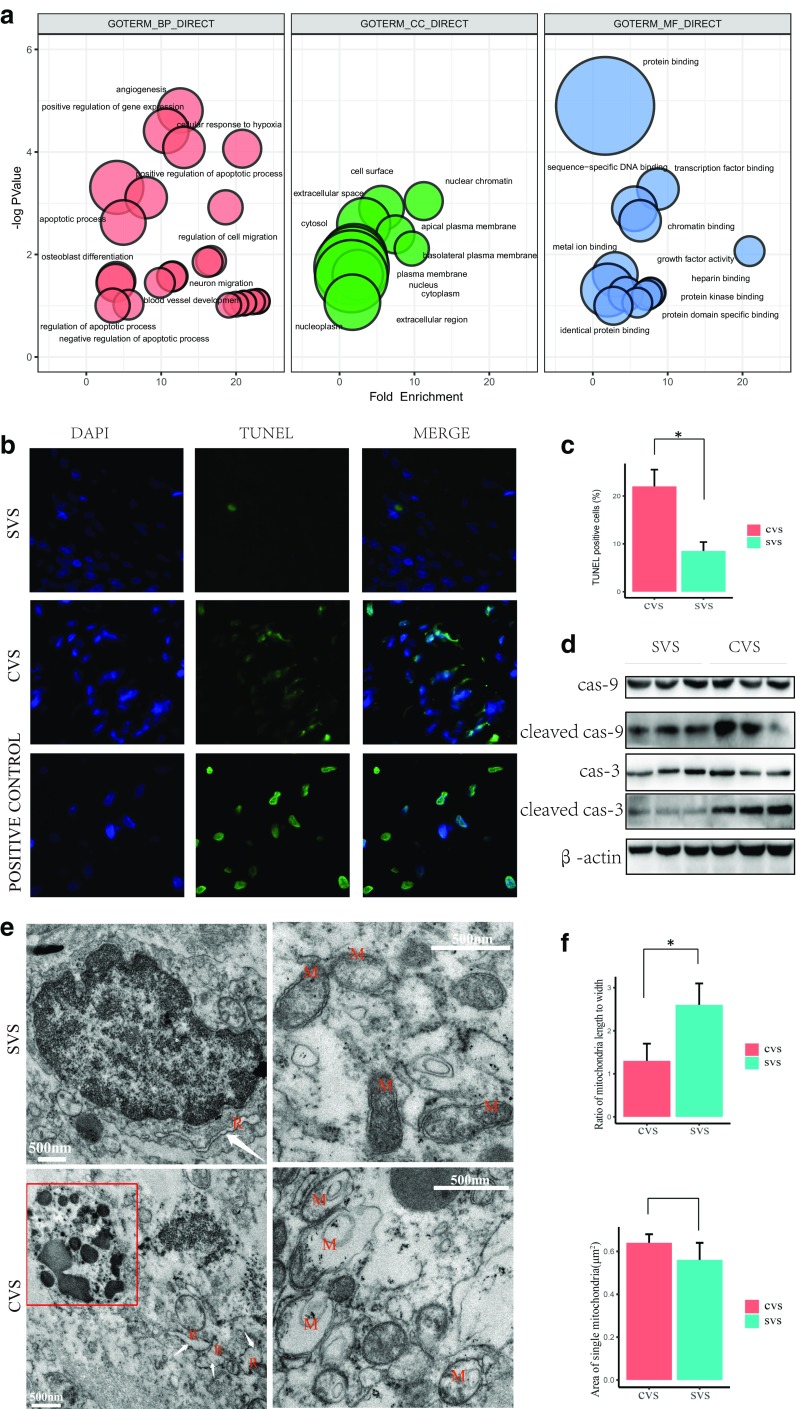
Biofunction enrichment analysis and verification. **a** Functional analysis of genes differentially regulated between CVS and SVS. –log10 of the enrichment *p* values for selected GO categories (BP biological process, CC cellular components, MF molecular function) are plotted relative to fold enrichments in each category. Circle size denotes the number of regulated genes. **b** Apoptosis was determined using TUNEL assay between CVS and SVS. Green fluorescence indicates TUNEL-positive cells in the microscopic fields. DAPI was used for nuclear staining (magnification × 400). **c** Quantification of apoptotic cardiomyocytes in two groups. Data are presented as the mean ± SEM, *n* = 3 in each group, *p < 0.05. **d** The protein expressions of caspase-3, caspase-9, cleaved caspase-3, and cleaved caspase-9 were determined by immunoblotting analysis. **e** Ultrastructural observations of CVS and SVS samples using transmission electron microscopy. R, endoplasmic reticulum (white arrow); M, mitochondria. Red rectangle point to apoptotic body. Scale bar, 0 .5μm. **f** The shape and area in CVS and SVS tissues were measured using ImageJ program. Data are presented as the mean ± SEM, *n* = 3 in each group, *p < 0.05

Gene ontology (GO) needs the co-differentially expressed DEmRNAs to be identified. With the DEmRNAs uploaded to the online tool DAVID, the DEmRNAs were significantly enriched in biological processes (BP), including angiogenesis, positive regulation of cell migration, cellular response to hypoxia, apoptosis, and neuroblast proliferation (Fig. 3a). For molecular function (MF), the DEmRNAs were involved in protein binding, transcription factor binding, platelet-derived growth factor receptor binding, sequence-specific DNA binding, chromatin binding, and fibronectin binding (Fig. 3a). In addition, the GO cell component (CC) analysis also showed that the DEmRNAs were significantly enriched in nuclear chromatin, cell surface, extracellular space, apical plasma membrane, and basolateral plasma membrane (Fig. 3a).

Further investigations were presented to determine the apoptosis, cellular response to hypoxia, and extracellular space changes in CVS tumor tissues by TUNEL. immunoblotting, and TEM. Compared with the SVS tissues, the CVS tissues showed less cellularity and increased apoptotic cells (TUNEL positive) (Fig. 3b and c), suggesting that apoptosis might be enhanced in CVS. Furthermore, apoptosis-related molecules expressions were determined by immunoblotting. The results showed that cleaved caspase-3 and cleaved caspase-9 expressions were increased in CVS tissues (Fig. 3d). Moreover, electron microscopy revealed ultrastructural signs of atypical cell death with larger and rounder mitochondria and the inner mitochondrial matrices were either damaged or almost absent, and dilatation of rough endoplasmic reticulum as well as the occurrence of apoptotic bodies in CVS (Fig. 3e and f). All of these results indicate increased degenerative changes in CVS.

### Susceptible pathways and clusters

**Fig. 4:**
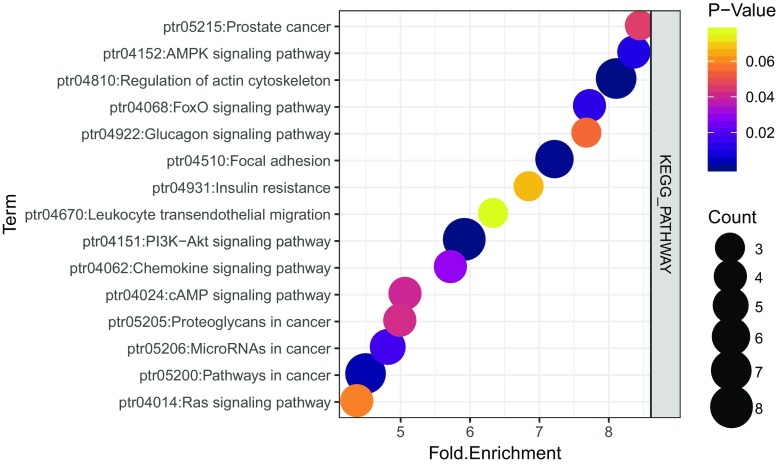
Kyoto encyclopedia of genes and genomes (KEGG) pathway enrichment analysis of the co-differentially-expressed DEmRNAs. Gene sets with top 10 values are plotted relative to normalized enrichment scores (NES). Circle size denotes the number of enriched genes in each category, and circle colors represent *p* values as indicated

The KEGG pathway map from DAVID indicated that the co-differentially expressed DEmRNAs were significantly enriched in the PI3K-Akt signaling pathway and the pathways in cancer, the AMPK, FOXO, and chemokine signaling pathways (Fig. 4a).

**Fig. 5:**
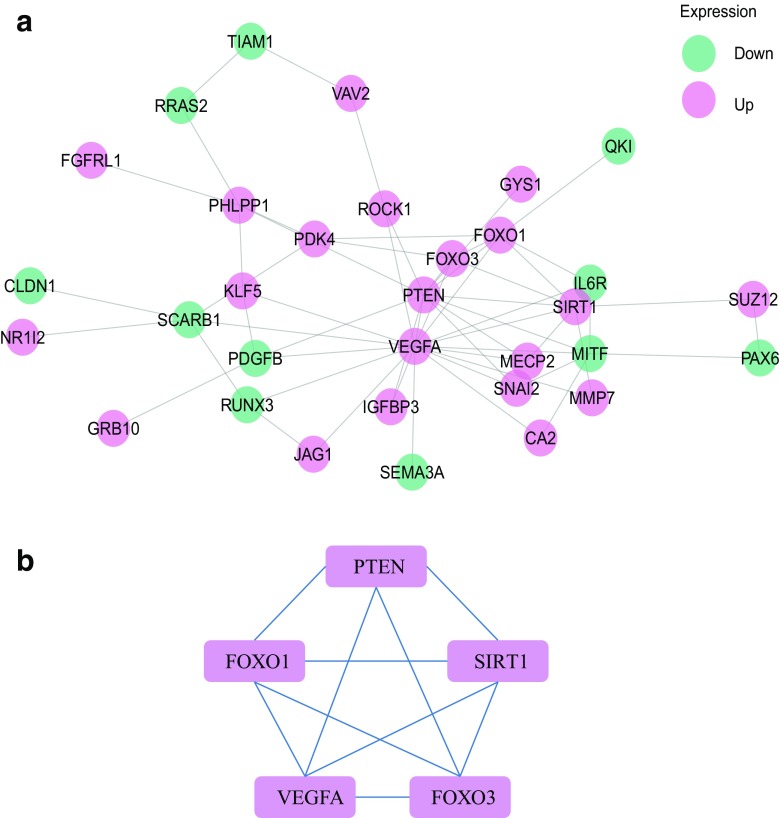
Protein–protein interaction (PPI). **a** PPI network between CVS and SVS. Pink, downregulated genes. Green, upregulated genes. **b** The most significant cluster from PPI network

Based on the PPI network derived from the STRING database, Cytoscape software was employed for visualizing the network of the co-differentially expressed DEmRNAs (Fig. 5a). The MCODE plugin in Cytoscape further presented the most significant cluster (Fig. 5B), including PTEN, FOXO1, FOXO3, VEGFA, and SIRT1. These hub genes were selected for further investigation.

### Clinical relevance of identified genes

**Fig. 6:**
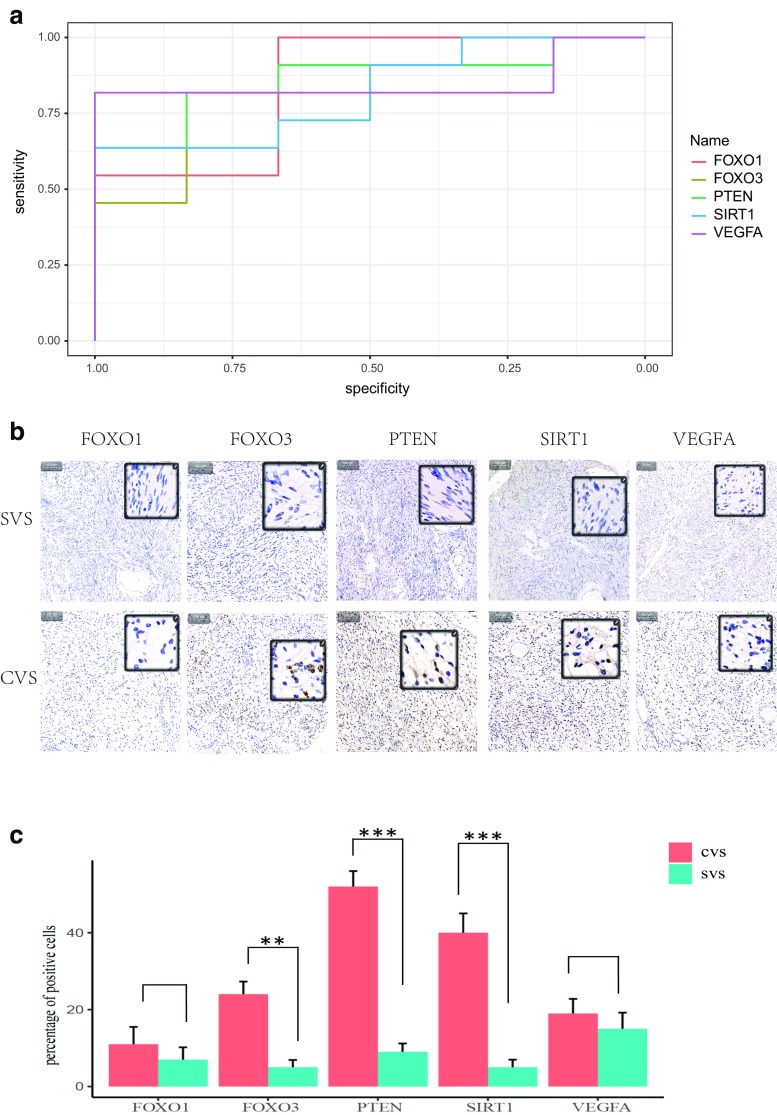
Clinical relevance of hub genes. **a** Receiver operating characteristic curve of FOXO1, FOXO3, PTEN, SIRT1, and VEGFA. **b** Immunohistochemical analysis showing levels of FOXO1, FOXO3, PTEN, SIRT1, and VEGFA in CVS tissues and SVS tissues. Scale bar, 100 μm. **c** Quantitative analysis of FOXO1, FOXO3, PTEN, SIRT1, and VEGFA expression in CVS and SVS tissues. Data are presented as the mean ± SEM, *n* = 3 in each group, **p < 0.01, ***p < 0.001

Further analysis of the hub genes in the most significant cluster in the SVS and CVS comparison is presented. The areas under the curve (AUC) of PTEN, FOXO1, FOXO3, VEGFA, and SIRT1 were 0.8636, 0.8485, 0.8333, 0.8182 and 0.8485, respectively, with the receiver operating characteristic curve (ROC) in Fig. 6a indicating that PTEN, FOXO1, FOXO3, VEGFA, and SIRT1 are potential biomarkers of CVS. Moreover, the immunohistochemistry demonstrated that PTEN, SIRT1, and FOXO3 were expressed at higher levels in CVS tissue (Fig. 6b), consistent with the cDNA microarray. Therefore, a combination of these three markers may potentially improve the predictions of disease outcome.

## Discussion

CVS has a shorter duration of symptoms and unpredictable biological behavior. However, the exact pathogenesis of the cystic formation within VS remains unclear. Previous researchers have analyzed cystic degeneration and enlargement from different perspectives, including histomorphology, cell distribution, and protein expression: (I) serum proteins exuded from a damaged blood-tumor barrier or protein secreted by tumor cells could result in cystic enlargement due to an osmotic effect (Schober et al. 1992); (II) Park et al. (Park et al. 2006) identified evidence such as hemosiderin-laden macrophages, hemosiderin deposits, and abnormal vessel proliferation in CVS, suggesting that previous bleeding within the lesion and repeated intratumoral microhemorrhage may lead to cystic formation; and (III) some reports (Moon et al. 2007) suggest the role of matrix metalloproteinases in the formation of cysts and adhesion of the tumor to the surrounding structures.

At present, there are few reports on the mechanism of the cystic changes of vestibular schwannoma from the perspective of genetics or transcriptomics. Our previous study showed that although the NF2 gene mutation is the major cause of VS, it may not directly participate in the cystic formation of VS (Zhang et al. 2014). In the present study, we obtained the lists of DEmRNAs and DEmiRNAs of CVS by cDNA microarray and miRNA sequencing, respectively, and acquired 1304 genes and 55 miRNAs after normalization. Comprehensive analysis of the miRNA-mRNA regulatory networks facilitates the understanding of gene regulation, providing further insight into the downstream effects of miRNA-mRNA. We combined all 55 DEmiRNAs with experimentally verified interactions of mRNA targets from miRTarBase and obtained 583 targeted mRNAs. Out of these targets, 540 were predicted mRNAs; however, only 43 were on the DEmRNAs list. We assumed that these 55 DEmiRNAs and 43 DEmRNAs may contribute to the cystic formation, and we constructed 67 DEmiRNA–DEmRNA pairs in more depth. miRNA is generally thought to negatively regulate its target gene by repressing mRNA translation or degrading mRNA. However, we observed a positive correlation in some networks of miRNA-mRNA, which is inconsistent with the intuitive understanding of the regulation mechanism of miRNA. The positive correlation may be the result of an adaptive response to changes in gene expression (Nunez et al. 2013).

GO and KEGG pathway analyses were performed to better understand the biological function of the co-differentially expressed DEmRNAs. The GO term analysis showed that these DEmRNAs were mainly involved in apoptosis, angiogenesis, cellular response to hypoxia, nuclear chromatin, cell surface, extracellular space, apical plasma membrane, and basolateral plasma membrane. In addition, the KEGG pathway analysis revealed that the co-differentially expressed DEmRNAs were significantly enriched in the PI3K-Akt signaling pathway and the pathways in cancer, the AMPK, FOXO, and chemokine signaling pathways. TUNEL and TEM were performed for further investigations on the apoptosis, cellular response to hypoxia, and extracellular space changes in CVS. It suggested that apoptosis might be enhanced in CVS. In addition, TEM revealed ultrastructural signs of cell death with the swelling of mitochondria and loss of cristae, dilatation and vesiculation of rough endoplasmic reticulum as well as the formation of vacuoles in the majority of cells, and the occurrence of apoptotic bodies and autophagosomes in CVS. Interestingly, the results obtained from the bioinformatics analysis are partially consistent with our experimental results, suggesting that apoptosis and oxidative stress may play a key role in the cystic changes of vestibular schwannoma. To a certain extent, managing these signaling pathways may facilitate the manipulation and prediction of cystic degeneration of VS.

The hub genes PTEN, FOXO1, FOXO3, VEGFA, and SIRT1 were recommended as useful biomarkers in the most enriched cluster in the SVS and CVS gene profile comparison. PTEN plays a key role in tumorigenesis as a tumor suppressor. PTEN has lipid phosphatase activity, which enables it to dephosphorylate the D3 position of phosphatidylinositol 3,4,5-triphosphate (PIP3) and antagonize the AKT signaling pathway. This signaling pathway is involved in a variety of physiological processes including cell growth, cell cycle progression, and survival. Previous study indicated that mutation of PTEN is associated with better clinical outcome in renal cell carcinomas with unclassified histology (Chen et al. 2016). Researchers found that PTEN hypermethylation values existed in VS (18% in cancer specimens vs. 0% in normal controls), but it cannot be correlated with observed clinical features (Lassaletta et al. 2006). VEGFA is a prototypic member of the VEGF protein family, which also includes VEGFB, VEGFC, VEGFD, and placental growth factor (PLGF). Moreover, VEGF has long been known as a molecular marker involved in VS development (Cayé-Thomasen et al. 2003; Koutsimpelas et al. 2012). Clinically, inhibitors of vascular endothelial growth factor are already utilized for VS treatment (Zhang et al. 2018) and biomarkers from DCE-MRI are predictive of VS volume response to inhibition of vascular endothelial growth factor inhibition (Li et al. 2016a). It is also reported that cross-talk between VEGFA and HGF signaling pathways in VS highlights growth factor associated pathway as an additional important therapeutic target (Dilwali et al. 2015). The FOXO family of transcription factors has important functions in controlling cellular homeostasis, with FOXO1, FOXO3, and FOXO4 being ubiquitously expressed (Eijkelenboom and Burgering 2013; Webb and Brunet 2014). Recent evidence supports the hypothesis that FOXO could influence the function of the nervous system and be of great significance for researching some neurodegenerative diseases. Actually, FOXO1 is an essential regulator of vascular growth that couples metabolic and proliferative activities in endothelial cells. Endothelial-restricted deletion of FOXO1 in mice induces a profound increase in endothelial cells proliferation that interferes with coordinated sprouting, thereby causing hyperplasia and vessel enlargement. Conversely, forced expression of FOXO1 restricts vascular expansion and leads to vessel thinning and hypobranching (Wilhelm et al. 2016). SIRT1 is a class III histone deacetylase that regulates many cellular functions, including cell proliferation, apoptosis, and inflammatory responses (Brooks and Gu 2009; Li 2013). Several studies have shown that SIRT1 may promote tumorigenesis of glioma (Qu et al. 2012; Li et al. 2016b). Additionally, Qu’s group identified that SIRT1 might be a promoter of glioma growth through the PTEN/PI3K/AKT signaling pathway (Qu et al. 2012).

## Conclusions

We compared the differences in gene expression between SVS and CVS and constructed miRNA–mRNA regulatory networks. The bioinformatics analysis revealed that the maps of apoptosis, cellular response to hypoxia, and the PI3K-Akt, AMPK, FOXO, and chemokine signaling pathway were significantly enriched. Of note, five hub genes including PTEN, FOXO1, FOXO3, VEGFA, and SIRT1 were identified, and they may contribute to the pathogenic and pathophysiological mechanisms of CVS formation. Further characterizing their biological functions and pathways may lift the veil of cystic formation at a molecular level and help to identify biomarkers for differential diagnosis and drug targets.

## Electronic supplementary material

Supplementary Figure 1 Magnetic resonance imaging of representative cystic and solid vestibular schwannomas. (PDF 8523 kb)

Supplementary Figure S2 t-stochastic neighbor embedding (t-SNE) of sample profiles (n_1_ = 12, n_2_ = 17) revealed several clusters. Green: CVS. Orange: SVS. (PDF 766 kb)
