# Supplementary figures and images for: Gene expression profiles between cystic and solid vestibular schwannoma indicate susceptible molecules and pathways in the cystic formation of vestibular schwannoma
Source: Funct Integr Genomics. 2019 Apr 5;19(4):673–84. doi: 10.1007/s10142-019-00672-5 (PMC6570702; doi:10.1007/s10142-019-00672-5)

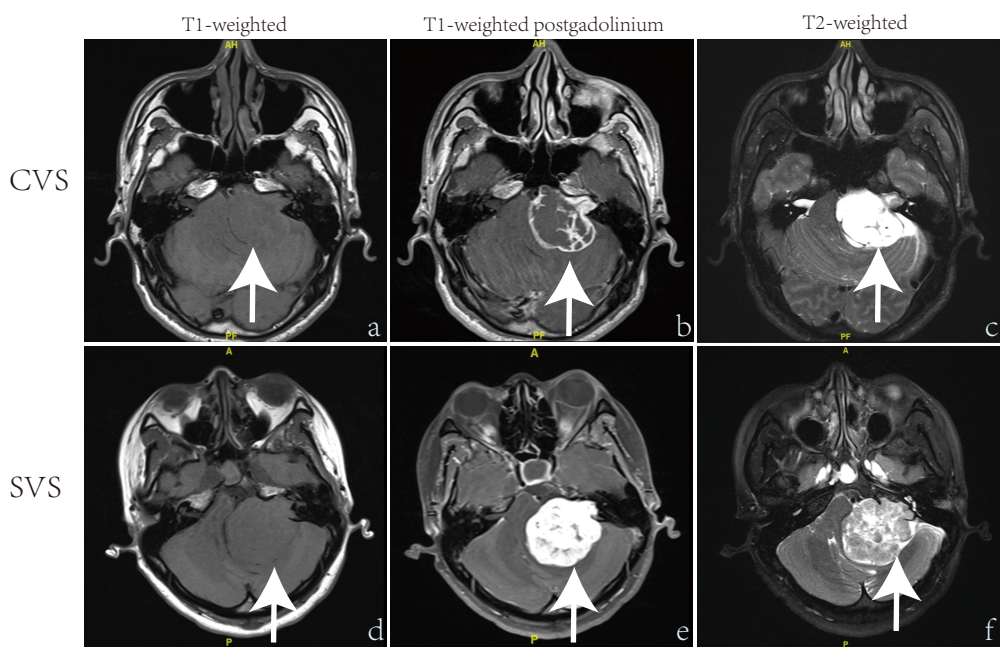

Supplement: Supplementary file 1 — Magnetic resonance imaging of representative cystic and solid vestibular schwannomas. (PDF 8523 kb) [file 10142_2019_672_MOESM1_ESM.pdf]

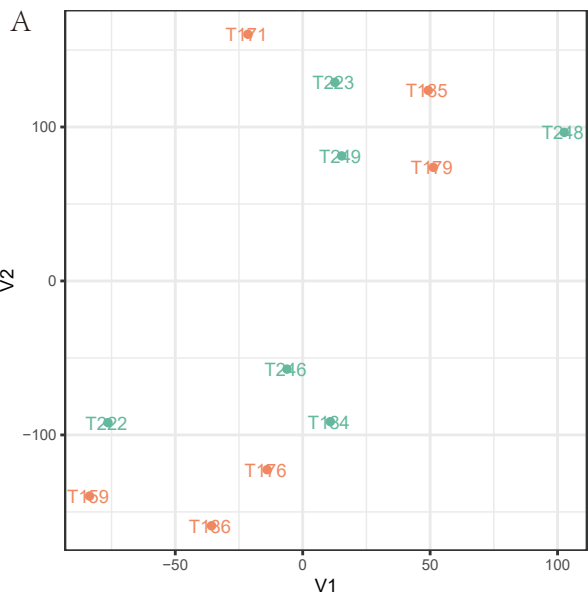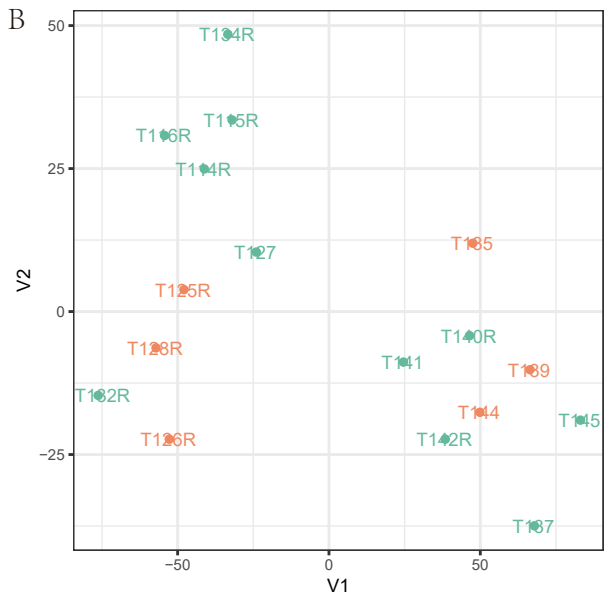

Supplement: Supplementary file 2 — t-stochastic neighbor embedding (t-SNE) of sample profiles (n1 = 12, n2 = 17) revealed several clusters. Green: CVS. Orange: SVS. (PDF 766 kb) [file 10142_2019_672_MOESM2_ESM.pdf]
